# White Sponge Nevus Caused by Keratin 4 Gene Mutation: A Case Report

**DOI:** 10.3390/genes13122184

**Published:** 2022-11-22

**Authors:** Yahui Qiao, Binjie Liu, Ruiqi Bai, Jingwen Cai, Qian Peng

**Affiliations:** 1 Xiangya Stomatological Hospital, Central South University, Changsha 410008, China; 2 Xiangya School of Stomatology, Central South University, Changsha 410008, China; 3 Hunan Key Laboratory of Oral Health Research, Central South University, Changsha 410008, China

**Keywords:** WSN, K4, gene mutation, genetic disorder, recurrence

## Abstract

White sponge nevus (WSN) is a rare autosomal dominant disease with a family history, often caused by mutations of the keratin 4 (K4) and keratin 13 (K13) genes in patients. It is characterized by frequently occurred white corrugated folds in the bilateral buccal mucosa with soft texture. On histopathological examination, hyperkeratosis of epithelial cells, edema, and vacuolar changes in the spinous cells are observed in the lesions, despite a normal layer of basal cells. WSN should be differentiated from other oral white spot diseases, mainly oral lichen planus, oral candidiasis, oral white edema, and Heck’s disease, to reduce misdiagnosis and unnecessary treatment. At present, there is no specific treatment method. The purpose of this study was to report the clinical data of four WSN patients of the same family with the K4 gene mutation. The occurrence of WSN in a pair of monozygotic twins with very similar clinical presentations was identified for the first time. The gene sequencing results showed that there was a heterozygous deletion (C. 438_440delCAA) in exon 1 of the K4 gene, resulting in an aspartic acid loss in both the proband and his father. Finally, the etiology, pathogenesis, pathological manifestations, clinical manifestations, diagnosis, differential diagnosis, and related treatment methods are discussed to provide a reference for clinical treatment of the disease.

## 1. Introduction

White sponge nevus (WSN) is an autosomal dominant disorder caused by mutations of the keratin 4 (K4) and keratin 13 (K13) genes, which usually occur in the oral mucosa [1]. With the features of white or grayish white corrugated folds or furrows in the shape of a “sponge”, WSN is often soft and painless. Sometimes the scales can disappear within a day and then reappear after 2 to 3 days. This disease is very rare, with an incidence of only 1/200,000 and has variable gene expression and irregular penetrance [1]. Thus far, a total of nine and ten pathogenic mutations have been identified in the K4 and K13 genes in patients with WSN, respectively. The same lesions can occur in areas other than oral mucosa, such as nose, esophagus, rectum, and genitalia [2]. The lesions may appear in infancy and become stable in adulthood. Clinically, WSN should be differentiated from other oral white spot diseases and a pathological biopsy and genetic analysis are necessary for a proper diagnosis. It is generally considered benign and therefore does not require special treatment for asymptomatic patients to reduce misdiagnosis and unnecessary treatment. However, treatment is recommended when the patient is symptomatic. The aim of this study was to report the occurrence of WSN in four members of the same family admitted to the Department of Oral Mucosa at the Xiangya Stomatological Hospital, Central South University, China. The patients included the proband, his father and twin sisters, whose very similar clinical presentations of the disease were first identified.

## 2. Case Report

**Figure 1:**
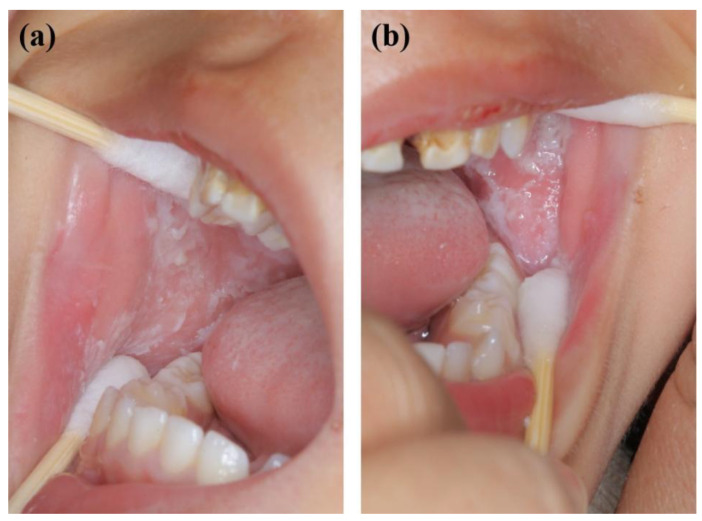
Oral manifestations of the proband (the 2-year-old boy) showing large gray and white folds and plaques in the (**a**) right and (**b**) left buccal mucosa.

**Figure 2:**
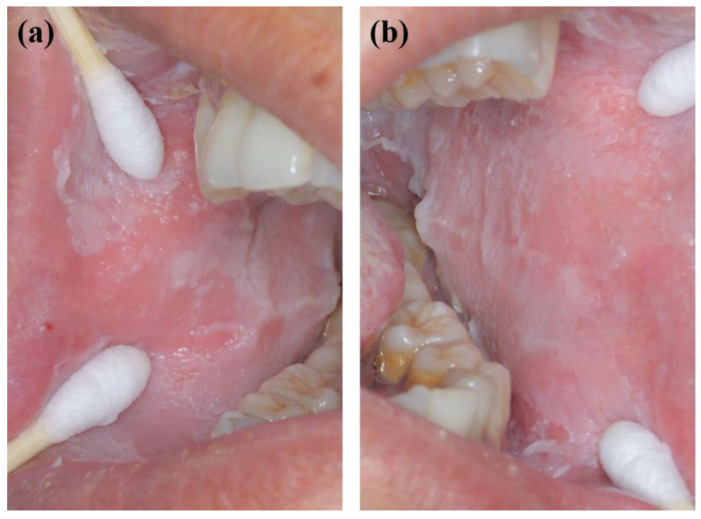
Oral manifestations of the father showing large gray and white folds and plaques in the right (**a**) and left (**b**) buccal mucosa.

**Figure 3:**
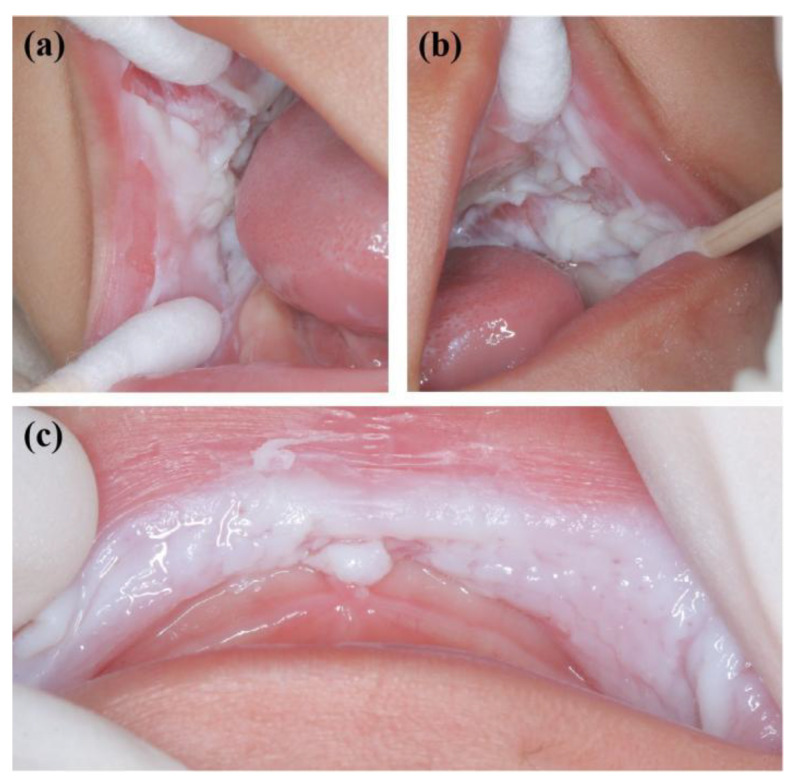
Oral manifestations of sister 1 showing large white corrugated folds in the (**a**) right and (**b**) left buccal mucosa and (**c**) inner mucosa of the upper lip, resembling a sponge.

**Figure 4:**
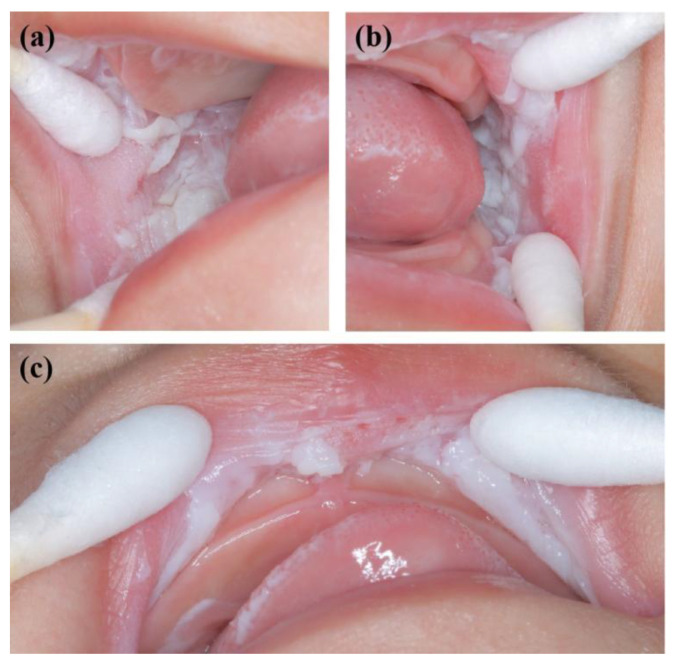
Oral manifestations of sister 2 showing large white corrugated folds in the (**a**) right and (**b**) left buccal mucosa and (**c**) inner mucosa of the upper lip, resembling a sponge.

**Figure 5:**
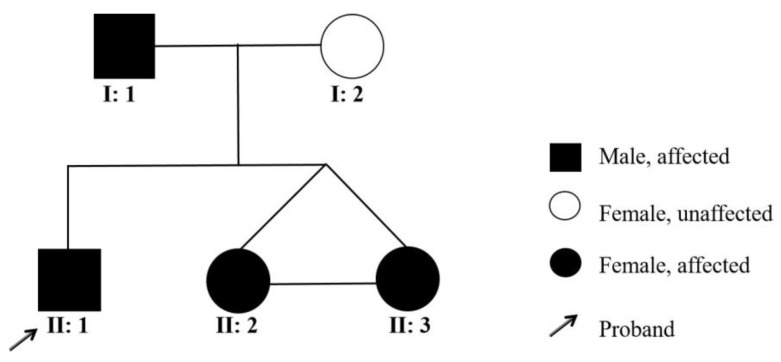
Pedigree of the proband’s family.

**Figure 6:**
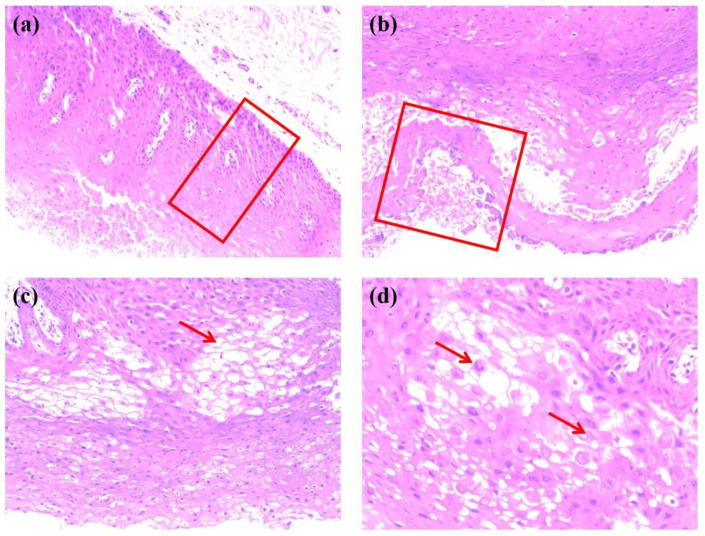
Hematoxylin-eosin staining of the right buccal mucosa of the proband’s father with (**a**) the red frame indicating enlarged spinous cells and well differentiated basal cells with increased number of layers (40×), (**b**) the red frame indicating incomplete keratinization of surface cells and uneven epithelial tissue with no inflammatory infiltration (100×), (**c**) the red arrow indicating vacuolar change of spinous cells (200×), and (**d**) the top and bottom arrows indicating spinous cells with nuclear pyknosis and cell edema, respectively (400×).

**Figure 7:**
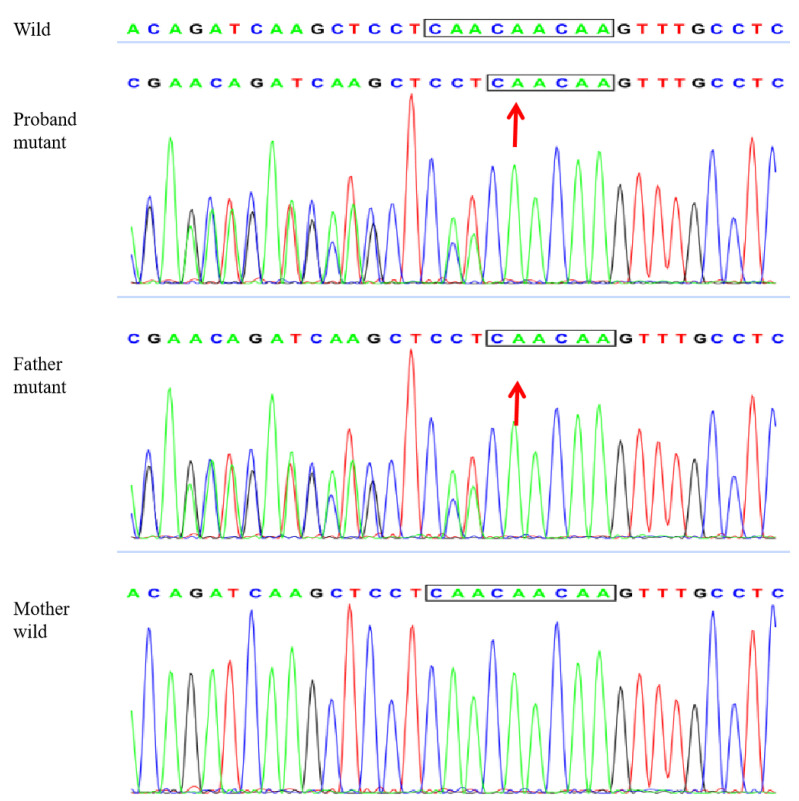
Gene sequencing results showing the heterozygous deletion (C. 438_440delCAA) in exon 1 of the K4 gene in the proband and his father, namely the aspartic acid loss, and no abnormality in the mother, with the arrows indicating the missing bases.

**Figure 8:**
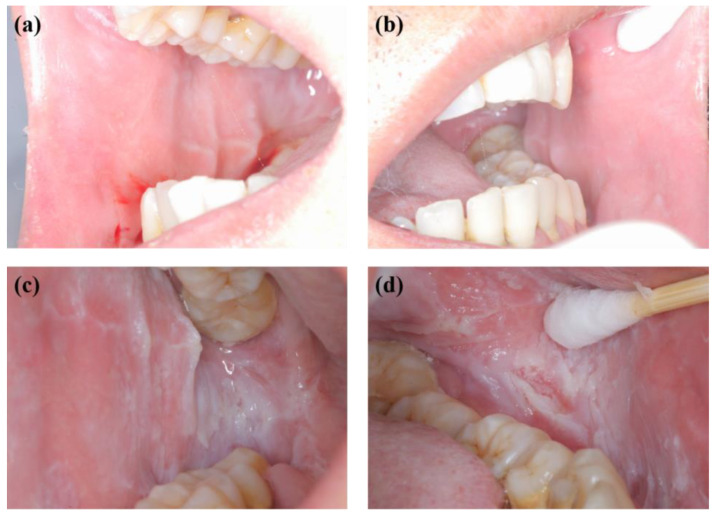
Oral mucosal examination for the proband’s father at follow-up showing significant reduction of lesions in the (**a**) right and (**b**) left buccal mucosa and a nearly complete disappearance of white corrugated plaques after one week and recurrence in the (**c**) right and (**d**) left buccal mucosa with greyish white corrugated folds and plaques after three months.

A 2-year-old boy, whose parents complained of “whitening of the buccal mucosa for 1 year”, came to the clinic for a consultation and treatment options. Since the incident, the child had no obvious symptoms. The clinical examination showed that his buccal mucosa had large creamy white folds with uneven thickness. The flaking mucosa could be removed after swabbing without bleeding and eroding the surface. There were no lesions in the tongue or mouth floor (Figure 1). As shown in Figure 2, Figure 3 and Figure 4, the father and twin sisters exhibited similar oral lesions. The patients denied having genital lesions. Because the father had no obvious symptoms in his oral mucosa, he had never consulted a doctor and had not received any treatment before this incident. The twin sisters had lesions since birth, with lesions in the same areas and thicker folds than their brother and father. The family pedigree is shown in Figure 5. It was initially diagnosed as WSN. The preliminary treatment plan was a biopsy combined with expectant management/pharmacological treatment. Considering the young age of the child, a histological examination was conducted on the intraoral lesion in his father’s mouth with his informed consent. The pathological findings included incompletely keratinized surface cells, enlarged, vacuolated and edematous spiny cells, and well-differentiated basal cells (Figure 6). According to the pathological diagnosis, WSN was confirmed in the buccal mucosa. For genetic testing, 4 mL of peripheral venous blood was collected from the proband, his father and his mother, respectively. The proband’s results were obtained by trio whole-exome sequencing and validated by paternal Sanger sequencing. The genetic sequences were not measured for the twin sisters because they were only six months old. The results showed a heterozygous deletion (C. 438_440delCAA) in the first exon of the K4 gene in the proband and his father, namely an aspartic acid loss, and no abnormality was found in his mother (Figure 7). The proband’s father was treated with topical applications of retinoic acid cream three times a day. One week later, the lesion area of bilateral buccal mucosa was significantly reduced and the white corrugated plaques basically disappeared (Figure 8a,b). After three months, however, the bilateral buccal mucosa was revisited with grayish white corrugated folds and plaques (Figure 8c,d). This observation verified the recurrence of WSN.

## 3. Discussion

The occurrence of WSN is associated with mutations in the K4 gene located on chromosome 12q13 or the K13 gene located on chromosome 17q21-q22 [3]. Keratin is the main structural protein of epithelial cells, forming cytoskeletal intermediate filaments. In addition to maintaining the integrity and mechanical stability of epithelial cells and tissues, some keratins also participate in intracellular signaling pathways, resisting stress and promoting wound healing and apoptosis [4,5]. The K4 and K13 genes have similar molecular structures, including a spiral rod-shaped region, a non-spiral head region, a tail region, and a connective region. The rod region has four α-helix fragments, including 1A, 1B, 2A, and 2B and three non-α-helix linkage regions, namely L1, L12, and L2, between the fragments [6]. Small changes in the sequence of these regions can lead to the formation of irregular intermediate filaments that disrupt their normal structure and function [7]. The damaged intermediate filaments may induce inflammation, which facilitates abnormal growth and division of epithelial cells, resulting in mucosal thickening. The occurrence of WSN reported in this study was caused by the heterozygous deletion in exon 1 of the K4 gene (C. 438_440delCAA), which was consistent with the previous report [8]. In fact, various mutation patterns of the K4 and K13 genes have been reported, as shown in Table 1 [5,9,10,11,12,13,14,15,16,17,18,19,20,21,22,23]. From the perspective of the mutation region, the K4 gene mutation occurs mainly in the 1A and 2B regions and sometimes in the non-coding region, while the K13 gene mutation occurs mainly in the 1A region and sometimes in L12 [6]. Similar cases have been reported in Japan and China.

Histologically, the lesions show significant thickening of the epithelium with well-defined layers, incomplete keratinization of surface cells, nuclei consolidation or disappearance, dispersion of keratinized hyaline grains in the superficial spinal layer, aggregation of keratin intermediate filaments in the upper spinal layer, and hyperplasia of well-differentiated basal cells. As shown in Figure 6, the pathological biopsy of the right buccal mucosa of the father demonstrated enlarged spinous cells with vacuolar changes and nuclear pyknosis, well-differentiated basal cells with more layers, incomplete keratinization of surface cells, and uneven epithelial tissue without inflammatory infiltration, conforming to the conventional pathological manifestations of WSN. In recent years, there have also been some pathological studies that revealed a positive granulocytic cytoplasmic phenotype for CD138 (syndecan-1) in the basal and suprabasal layers, a small infiltration of inflammatory cells in the connective tissue, and edema and breakage of collagen fibers [24]. It is also believed that the disorder of epithelial surface shedding in the lesion area is associated with the abnormal function of the membrane pericytes (Odland vesicles), with insufficient intercellular pericytes and increased bridging grains. As a result, there will be accumulation of epithelial surface cells, which is responsible for the spongy appearance. The white color is caused by the high proliferation of saliva-induced terminally differentiated keratogenic cells [8].

WSN is typically characterized by white, painless, and spongy plaques often occurring in the buccal, oral base, lingual, abdominal, or sometimes palatal and gingival mucosae. It may also occur in the mucosae of the nose, esophagus, rectum, and genitalia without the coverage of keratinizing squamous epithelium [25,26]. The clinical manifestations are gray and white corrugated folds or furrows, in the shape of grass or wrinkled paper, in a special pearlescent color or bright pink. The uneven surface may be present as small follicles with a soft texture. The mucous membrane can be removed with no pain or bleeding after swabbing. There are usually no obvious symptoms, but the change in mucosal texture and appearance may have a psychological impact on the patient. In addition, a few people may feel a roughness, a burning pain, or a loss of taste. There is no gender or racial difference. It often occurs in children and adolescents and is often unnoticed. It develops rapidly in adolescence and gradually becomes stable in adulthood [3,8,27]. WSN plaques are generally considered benign. However, they may become malignant with the influence of living habits [23]. In this study, large areas of painless spongy white plaques appeared on both sides of the buccal mucosae of the proband and his father and on both sides of the buccal mucosae and upper lips of the twin sisters. The monozygotic twins had extremely similar symptoms, characterized by the same plaque position and thicker folds compared with the proband and father. It may be attributed to the similarities of twin genes, dietary structure, and living habits.

WSN is usually diagnosed based on clinical presentation and a positive family history. For disseminated cases, a combination of pathological diagnosis and genetic analysis is often required to further confirm the diagnosis. In this study, the WSN was diagnosed based on clinical presentation, pathological features, and gene sequencing results. Clinically, WSN should be differentiated from other oral white spot diseases, mainly including oral lichen planus, oral candidiasis, oral white edema, and Heck’s disease (Table 2) [3,28,29]. Other diseases such as congenital thick nail disease, follicular keratosis, lupus erythematosus, secondary syphilis, condyloma acuminata, dyskeratosis congenita, and squamous cell carcinoma may also have similar localized oral lesions [29]. They can generally be identified based on the clinical manifestations and systemic symptoms accompanying these diseases [24,30].

Although WSN is prone to recurrence, it is generally considered benign and therefore does not require special treatment for asymptomatic patients. At present, the overall performance of various treatments is not good although some studies have reported certain clinical efficacy after pharmacological treatment with vincristine, chlorhexidine, penicillin, tetracycline, azithromycin, clotrimazole lotion, or local surgical excision (Table 3) [1,2,11,14,20,21,23,31,32,33,34,35,36,37,38,39,40,41]. However, most of them showed signs of recurrence. In this report, the proband’s father applied vitamin A acid cream, which affected mitosis and epidermal cell renewal, keratinization, and epithelial metabolism [36], after the biopsy. One week later, the outpatient review showed that the lesion area of the bilateral buccal mucosa was significantly reduced and the white corrugated plaques basically disappeared. Three months later, the bilateral buccal mucosa redeveloped grayish white corrugated folds and plaques (Figure 8). The positive initial effect and subsequent recurrence in the later stage were consistent with the majority of WSN treatment experiences. According to previous reports, when misdiagnosed as candidiasis and treated with antifungal therapy, there was no effect. On the other hand, when misdiagnosed as lichen planus, the lesion expanded after topical application of steroid ointment for two weeks [2,28]. When patients with WSN have a burning sensation or other symptoms, dissimilar treatment modalities can be selected according to different conditions. If the patients have no obvious symptoms but only complain of changes in the texture and appearance of the oral mucosa, treatment is generally not necessary, and they should be instructed to maintain good oral hygiene and regular follow-up.

## 4. Conclusions

This paper presented a case of occurrence of WSN in four members of the same family caused by the K4 gene mutation (c.438_440delCAA). The occurrence of WSN in monozygotic twins with very similar clinical presentations was confirmed for the first time. A detailed discussion of the etiology, pathogenesis, pathological manifestations, clinical manifestations, differential diagnosis, and treatment of WSN was also provided to guide its diagnosis and treatment. The mutation mechanism and inheritance mode of keratin gene mutations are not completely clear and there is no specific treatment method, which needs further research. To reduce missed diagnosis, misdiagnosis, and mistreatment, it is necessary to pay more attention to related medical history and family history, along with careful physical examination and biopsy.

## Figures and Tables

**Table 1:** Types of mutations of the K4 and K13 genes in patients with WSN.

Gene	Base Mutation	Amino Acid Changes	Chromosome Location	Region	Patients				Country/Area	Reference
					Gender	Age	Number of Family Morbidity/Number of Family Members	Family Patients		
K4	419–420, with the insertion of ACA	Frame shift	12q.13.13	1A	−	−	−	−	−	[5]
	436–438, with the deletion of AAC	Asp 145 del	12q.13.13	1A	M	−	4/6	Father, sister 1, and sister 2	−	[9]
	438–440, with the deletion of CAA	Asp 146 del	12q.13.13	1A	M	2	4/5	Father, sister 1, and sister 2	China	This study
	458–459, with the insertion of ACA	Glu add	12q.13.13	1A	F	27	3/4	Mother and brother	Italy	[10]
	478–480, with the deletion of CAA	Asp 160 del	12q.13.13	1A	M	11	4/8	Grandmother *, mother, and brother	Japan	[11]
	G1345A	Glu449 →Lys	12q.13.13	2B	M	23	2/6	Father	China	[12]
	G1558A	Glu520→Lys	12q.13.13	2B	M	13	-	Father *	Japan	[13]
	G1829A	Glu520→Lys	12q.13.13	2B	F	32	4/6	Mother, sister, and daughter	China	[14]
	A2324G	−	12q.13.13	Non- coding area	F	32	4/6	Mother, sister, and daughter	China	[14]
K13	T323C	Met108 →Thr	17q21.2	1A	M	−	8/12	Grandmother *, dad *, aunt *, cousin 1 *, cousin 2 *, son 1 *, and son 2 *	England	[15]
	T332C	Leu111 →Pro	17q21.2	1A	F	36	−	Father *, sister *, daughter, son, and nephew *	Japan	[16]
	A335G	Asp112 →Ger	17q21.2	1A	M	18	18(9 *)/43	-	Scotland	[17]
	C340T	Arg114 →Cys	17q21.2	1A	M	−	5/11	Grandmother, mother, aunts, and cousins	China	[18]
	G341A	Arg114 →His	17q21.2	1A	F	48	−	No family history	Japan	[19]
	T344G	Leu115→Arg	17q21.2	1A	F	19	3/5	Mother and sister	Korea	[20]
	T344C	Leu115 →Pro	17q21.2	1A	F	−	−	No family history	Denmark	[15]
	T352G	Tyr118→Asp	17q21.2	1A	M	36	21(11 *)/55	−	Turkey	[21]
	T356C	Leu119 →Pro	17q21.2	1A	F	−	7/16	−	Italy	[22]
	1023–1077 del	Lys342-GIn359 del	17q21.2	L12	F	−	12/35	−	Netherlands	[23]

* Similar lesions without confirmation.

**Table 2:** Differential diagnosis between WSN and oral lichen planus, oral candidiasis, oral white edema, and Heck’s disease [3,28,29].

Differential Diagnosis	Clinical Presentations	Pathological Manifestations
Oral lichen planus	Being prevalent in middle-aged women, often with symmetrical lesions; white or grayish white linear, reticular, dendritic, ring-like, or semi-ring-like papules that remain after scraping; self-conscious mucosal roughness, burning sensation, and tingling; features of typical skin lesions including flat polygonal papules and Wickham pattern	Thin keratin layer; lightly proliferated or atrophied spiny layer, without vacuolation; liquefied and degenerated basal cell layer; dense lymphocyte infiltration in the lamina propria in the form of bands; occasional abnormal epithelial hyperplasia
Oral candidiasis	Scattered distribution of small spots (pseudomembranous candidiasis) and patches of milky white elevations (chronic hyperplastic candidiasis) in the oral cavity; visible red trauma after removal; no family history	Microscopic examination showing spores and pseudohyphae
Oral white edema	Transparent, grayish white, smooth, and borderless “veil-like” film, mostly occurring in the premolar and molar occlusal lines, with soft texture and no pressure pain; temporary disappearance of white edema in the mucosal stretching test; having a relationship with local irritation factors such as smoking, alcohol, oral mucosal trauma, and other local irritation factors	Epithelial thickening; intraepithelial cell edema; nuclear consolidation or disappearance; vacuolation; no keratinization or incomplete keratinization of the superficial layer
Heck’s disease	Multiple, round, white, or flesh-colored papules or nodules in the oral mucosa	Acanthosis, variable papillomatosis, para parakeratosis, hyperkeratosis, rete ridge elongation, and perinuclear halos

**Table 3:** Clinical manifestations and treatment modalities of WSN.

Family/Genetic History	Clinical Presentation			Treatment			Reference
	Proband	Symptoms	Locations	Modalities and Medications	Time	Effect	
	Gender and Age						
Yes (grandmother, mother, and brother)	M 11	White lesions	Bilateral buccal mucosa and tongue lateral edge	−	−	−	[11]
Yes (mother)	M 15	White and spongy lesions	Bilateral buccal mucosa	Mouthwash using 0.12% chlorhexidine (5 mL b.i.d.)	8 days	Fading and having recurrence in 1 month after discontinuation	[31]
Yes (grandmother, father, brother, and daughter)	F 27	White, thickened, folded, and velvet-like plaques, with no scratches	Bilateral buccal mucosa, tongue mucosa, and genital	Local application of hydrocortisone	−	No improvement	[32]
Yes (brother)	M 55	White, follicular, soft, unevenly thickened, rough, and corrugated plaques; being removable after scraping; no smoking history	Bilateral ventral tongue, bilateral buccal mucosa, and lower lip mucosa	Biopsy of the right buccal lesion; mouthwash using antibiotics and compound chlorhexidine gargle after surgery	−	Shrinking	[33]
Yes (father and brother)	F 13	White, corrugated, diffuse, soft, and thickened plaques	Bilateral buccal mucosa, soft palate, tongue dorsum, lower lip mucosa, and hard palate	No treatment	−	−	[1]
Yes (mother, sister, and daughter)	F 32	White, soft, thickened, and folded plaques	Bilateral buccal mucosa and lip mucosa	No treatment	−	−	[14]
Yes (mother and sister)	F 19	White, corrugated, spongy, thickened, and fissured plaques	Bilateral buccal mucosa, lip mucosa, gingiva, palate, tongue mucosa, and mouth floor mucosa	No treatment	−	−	[20]
Yes (21 people)	M 36	Corrugated and partially fibrous elevation; spongy and diffuse plaques; fissured tongue; feeling painful after eating spicy and irritating food	Bilateral buccal mucosa, ventral tongue mucosa, sulcus vestibularis, and mouth floor mucosa	No treatment	−	−	[21]
Yes (12 people)	F −	White, spongy, and rough plaques	Oral mucosa, mouth floor mucosa, tongue side, vagina, and cervix	No treatment	−	−	[23]
Yes (mother and the eldest daughter)	F 47	White, corrugated, and folded plaques	Bilateral buccal mucosa, lip mucosa, and tongue mucosa	No treatment	−	−	[34]
Yes (father)	M 13	White lesions	Bilateral buccal mucosa	No treatment	−	−	[35]
No	M 22	White, spongy, folded, and rough plaques	Bilateral buccal mucosa, lip mucosa, and bilateral lingual edges	Local application of steroid ointment	2 weeks	Expanding	[2]
				Oral administration using azithromycin (after biopsy)	−	Shrinking	
				Local application of tetracycline ointment	2 weeks	Shrinking and having recurrence after discontinuation	
				Oral administration using multivitamin	6 months	Complete disappearance of lesions and no recurrence	
No	M 8	Grayish white, folded, soft, clearly delineated, and bead-like plaques of about 2 to 3 mm in diameter	Bilateral buccal mucosa and lip mucosa	Local application of vitamin A acid	2 months	Fading and no recurrence for six months after discontinuation	[36]
No	M 32	White and spongy plaques	Bilateral buccal mucosa and lip mucosa	Oral administration using ampicillin (250 mg q.d.s.)	2 weeks	Shrinking and having recurrence after 10 weeks	[37]
No	M 69	Dry mouth (in hospital with bronchitis)	Bilateral buccal mucosa, tongue mucosa, and lip mucosa	Oral administration using ampicillin (250 mg q.d.s.)	−	Fading and having recurrence after discontinuation	[38]
				Oral administration using tetracycline (250 mg t.i.d.)	Several days	Fading and having recurrence after discontinuation	
No	F 51	Oral pain for 30 years	Oral mucosa and vulva	Mouthwash using 0–25% tetracycline solution (1 q.d.)	-	Fading and having pain relief	
No	F 18	Burning sensation; irregular shape; clear boundary; non-scratchable plaques	Bilateral buccal mucosa	Mouthwash using 1% clotrimazole (5 q.d.)	1 month	Fading	[39]
No	M 50	White, soft, and rough plaques	Tongue mucosa	CO_2_ laser resection of lesion	-	Having recurrence	[40]
				Surgical resection	2 years	No recurrence	
No	M 46	White and soft plaques and no peeling off after scratching	Tongue mucosa and lateral edge	Local application of 0.1% tretinoin acetate and 0.1% retinoic acid	2 months	No improvement	[41]
				Oral administration using doxycycline (100 mg.d)	6 weeks	Having improvement and stable condition after six months	

## Data Availability

The data presented in this study are available on request from the corresponding author.
